# Inhaled milrinone in cardiac surgical patients: a pilot randomized controlled trial of jet vs. mesh nebulization

**DOI:** 10.1038/s41598-020-58902-x

**Published:** 2020-02-07

**Authors:** Anne Quynh-Nhu Nguyen, André Y. Denault, Yves Théoret, Louis P. Perrault, France Varin

**Affiliations:** 1 Faculty of Pharmacy, Université de Montréal, Montreal, Canada; 2 Department of Anesthesiology and Critical Care Division, Montreal Heart Institute, Université de Montréal, Montreal, Canada; 3 Clinical Pharmacology Unit, CHU Sainte-Justine, Montreal, Canada; 4 Department of Cardiac Surgery, Montreal Heart Institute, Université de Montréal, Montreal, Canada

**Keywords:** Atherosclerosis, Vascular diseases, Predictive markers, Phase II trials, Risk factors

## Abstract

Inhaled milrinone administered before cardiopulmonary bypass (CPB) reduces the severity of pulmonary hypertension during cardiac surgery. However, milrinone pharmacokinetics has not been determined for this route of administration. The objective of this study was to investigate inhaled milrinone dosing *in vitro* and early plasma concentrations *in vivo* after jet and mesh nebulization. Twelve pulmonary hypertensive patients scheduled for cardiac surgery were randomized to receive milrinone (5 mg) by inhalation before CPB using a jet or mesh nebulizer. *In vitro* experiments were conducted to determine the inhaled dose delivered with either jet or mesh nebulization. *In vivo* experiments involved hemodynamic monitoring and blood samples drawn from patients for the first 15 min after the end of inhalation to determine early plasma concentrations. After mesh nebulization, the mean *in vitro* inhaled dose was almost 3-fold higher compared to jet nebulization (46.4% vs 16.6% for mesh and jet, respectively; mean difference, 29.8%; 95% CI, 14.1 to 45.5; *P* = 0.006). Consistent with this, the early plasma concentrations *in vivo* were also 2–3 fold higher after mesh nebulization (*P* = 0.002–0.005). After inhalation (jet or mesh nebulization), milrinone early plasma concentrations remained within the therapeutic range. No systemic hypotension was reported in our patients.

## Introduction

Intravenous milrinone, a phosphodiesterase inhibitor and inodilator, has been extensively used in cardiac surgery for the treatment of pulmonary hypertension (PH), particularly during difficult separation from cardiopulmonary bypass (CPB)^1–4^. An important drawback of intravenous milrinone is its association with systemic hypotension^5–7^. To avoid this side effect, inhalation has been proposed as an alternative therapeutic route of administration for milrinone^8–10^. Pulmonary drug delivery presents advantages such as rapid absorption, high bioavailability, and high local concentrations^11^. Consequently, a hypothesis was put forward in the past decade that inhaled milrinone administered prior to CPB would have a protective effect against the exacerbation of PH in cardiac surgical patients^12,13^ by minimizing CPB-related inflammation^14^, preventing pulmonary endothelial dysfunction^15^, and facilitating separation from CPB^16^. More recently, a multicenter randomized controlled trial demonstrated the clinical efficacy of inhaled milrinone in reducing the degree of PH; however, it was not associated with a reduction in difficulty separating from CPB^17^. Several factors could explain those results, including suboptimal drug delivery^18^.

Nebulizers are commonly used drug delivery devices in aerosol therapy for patients with pulmonary diseases. These devices operate by converting liquid formulations into fine breathable droplets. There are three types of nebulizers: jet, ultrasonic and mesh^19^. Apart from four clinical studies using ultrasonic^9,14^ or mesh nebulizers^17,20^, jet nebulizers have been the standard drug delivery devices for inhaled milrinone in adult cardiac patients. Jet nebulizers use a high velocity jet of compressed gas (air or oxygen) flowing through the solution to draw and shear it into aerosol droplets of different range of sizes. Ultrasonic nebulizers use a high frequency vibrating piezoelectric element to produce ultrasonic waves into the solution and breaking it up at the surface into small aerosol droplets. Improvements in nebulization technologies have led to the development of mesh nebulizers using a multiaperture vibrating mesh (up to 10 000) to force the solution through the holes and generate a high fraction of fine aerosol droplets^21,22^. Mesh nebulizers have gained increasing popularity over the past decade, especially for delivering aerosolized drug therapy to mechanically ventilated patients. They offer many advantages over the two other types of nebulizers – i.e., portability due to a compact design, no secondary airflow, no liquid heating, and high efficiency at delivering drugs to the lungs with little wasted drug^23^.

This report on inhaled milrinone presents results obtained from a randomized controlled pilot study in cardiac surgical patients undergoing CPB. The objectives of this study were twofold: first, to assess the early plasma concentrations of milrinone in patients after both types of nebulization; and second, to verify whether the systemic exposure *in vivo* was consistent with the inhaled dosing *in vitro*. It was hypothesized that given the smaller particle size, milrinone delivered with mesh nebulization would generate higher early plasma levels compared to jet nebulization.

## Results

Twelve cardiac surgical patients were recruited between December 2006 and June 2007, with similar characteristics between both groups (Table 1).

**Table 1:** Patient Characteristics.

	Nebulizer	
	Jet (n = 6)	Mesh (n = 6)
Gender (f : m)	5 : 1	2 : 4
Age (yr)	65 (9)	74 (8)
Weight (kg)	76 (20)	73 (23)
**Surgical procedure**		
CABG	1	0
Single valve	2	1
Complex	2	4
Other	1	1

All values are mean (standard deviation). CABG = coronary artery bypass grafting.

In the present study, hemodynamic improvement for each parameter including mean arterial pressure (mAP), mean pulmonary artery pressure (mPAP) and the mAP/mPAP ratio refers to the maximum response after inhalation in variation from baseline (ΔEmax = post-inhalation value – baseline value). Within and between group comparisons are presented in Table 2. The administration of milrinone using mesh nebulization significantly reduced mPAP by 26.2% (26.4 vs. 19.3 mmHg for baseline and post-inhalation, respectively; mean difference, −7.1 mmHg; 95% confidence interval [CI], −10.8 to −3.3, *P* = 0.005) and increased the mAP/mPAP ratio by 32.1% (2.6 vs. 3.5 for baseline and post-inhalation, respectively; mean difference, 0.8; 95% CI, 0.4 to 1.3, *P* = 0.005). These hemodynamic improvements were significant after mesh nebulization, but not statistically significant after jet nebulization. No systemic hypotension was reported in our patients.

**Table 2:** Hemodynamic Monitoring in Cardiac Surgical Patients after Jet (n = 6) and Mesh (n = 6) Nebulization.

		Nebulizer						*P*-value
		n	Jet	*P*-value	n	Mesh	*P*-value	
				Within			Within	Between
mAP	Baseline (mmHg)	6	82.6 (10.2)		6	67.4 (5.3)		0.009*
	Post-inhalation (mmHg)	5	77.6 (15.9)		6	65.0 (8.9)		0.13
	ΔEmax	5	−2.4 (19.3)	0.80	6	−2.4 (10.7)	0.60	1.00
mPAP	Baseline (mmHg)	6	30.3 (11.5)		6	26.4 (5.1)		0.47
	Post-inhalation (mmHg)	5	25.8 (3.7)		6	19.3 (4.0)		0.02*
	ΔEmax	5	−6.1 (9.3)	0.21	6	−7.1 (3.6)	0.005*	0.81
mAP/mPAP	Baseline	6	3.0 (1.0)		6	2.6 (0.5)		0.44
	Post-inhalation (mmHg)	5	3.1 (1.1)		6	3.5 (0.8)		0.55
	ΔEmax	5	0.4 (0.4)	0.10	6	0.8 (0.4)	0.005*	0.11

All values are mean (standard deviation). ΔEmax values for each parameter are expressed as the maximum response after inhalation in variation from baseline. Measurements are post-induction of anesthesia. *P < 0.05. Emax = maximum effect; mAP = mean aterial pressure; mPAP = mean pulmonary artery pressure.

### *In vivo* early systemic exposure

**Figure 1:**
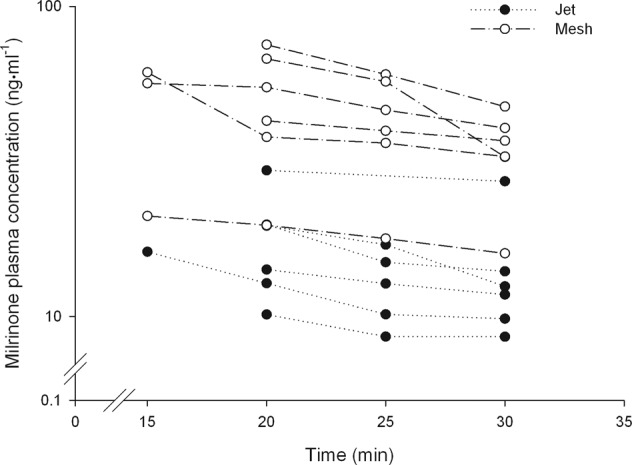
Milrinone plasma concentration-time profiles for 12 cardiac surgical patients after limited sampling (n = 4) following the administration of a 5 mg dose using jet or mesh nebulization.

Milrinone mean plasma concentrations and individual concentration-time profiles are presented in Table 3 and Fig. 1, respectively. Overall, early systemic exposure of milrinone was significantly higher (2–3 fold) using mesh nebulization.

**Table 3:** Milrinone Plasma Concentrations in Cardiac Surgical Patients after Jet (n = 6) and Mesh (n = 6) Nebulization.

Time after start of inhalation	Nebulizer				*P*-value
	n	Jet	n	Mesh	
		Concentration		Concentration	
(min)		(ng·ml^−1^)		(ng·ml^−1^)	
15	1	16.1 (n/a)	3	46.3 (21.9)	n/a
20	6	17.7 (7.0)	6	49.7 (20.5)	0.005*
25	5	12.7 (3.4)	6	42.9 (15.5)	0.002*
30	6	14.0 (6.8)	6	34.4 (10.6)	0.003*

All values are mean (standard deviation). *P < 0.05. n/a = not applicable.

### *In vitro* inhaled dose

Results for total dose recovered from *Setting 1* and *Setting 2* are summarized in Table 4. In *Setting 1*, the mean percentage emitted dose (filter A) was similar with both types of nebulizers (64.0 vs. 68.0% for jet and mesh, respectively; mean difference, 4.1%; 95% CI, −5.4 to 13.5, *P* = 0.30). However, distribution of residual dose and wasted dose differed within the nebulizer components (cup and T-piece). Residual dose in the nebulizer cup was greater after jet nebulization (29.7 vs. 3.1% for jet and mesh, respectively; mean difference, 26.7%; 95% CI, 24.0 to 29.3; *P* < 0.001). On the other hand, wasted dose in the nebulizer T-piece was greater after mesh nebulization (1.0% vs. 25.4% for jet and mesh, respectively; mean difference, 24.4%; 95% CI, 15.1 to 33.6; *P* = 0.004). Mean total dose recovered was 94.7% and 96.5% of nominal dose (5 mg) with jet and mesh nebulization, respectively (mean difference, 1.7%; 95% CI, −3.0 to 6.5; *P* = 0.37) and a high degree of consistency (CV%, 2.1% and 2.3%, respectively).

**Table 4:** *In Vitro* Experiments for Milrinone Dose Recovery.

	Nebulizer				∆	95%CI		*P*-value
	n	Jet	n	Mesh		Lower	Upper	
***Setting 1***								
Emitted dose (filter A)	3	64.0 (0.5)	3	68.0 (5.9)	4.1	−5.4	13.5	0.30
Residual dose (nebulizer cup)	3	29.7 (1.6)	3	3.1 (0.2)	−26.7	−29.3	−24.0	<0.001*
Wasted dose (nebulizer T-piece)	2	1.0 (0.2)	3	25.4 (3.9)	24.4	15.1	33.6	0.004*
Total dose recovered		**94.7 (2.0)**		**96.5 (2.3)**	1.7	−3.0	6.5	0.37
***Setting 2***								
Inhaled dose (filter B)	3	16.6 (1.7)	3	46.4 (9.6)	29.8	14.1	45.5	0.006*
Exhaled dose (filter C)	3	34.1 (4.8)	3	7.4 (0.2)	−26.7	−34.5	−19.0	<0.001*
Residual dose (nebulizer cup)	3	27.0 (0.6)	3	3.1 (0.2)	−24.0	−24.9	−23.0	<0.001*
Wasted dose (nebulizer T-piece)	3	0.9 (0.1)	3	18.2 (4.4)	17.3	10.3	24.3	0.002*
Total dose recovered		**78.6 (5.8)**		**75.1 (5.7)**	−3.5	−16.4	9.4	0.49
Unrecovered dose (Y-connector + endotracheal tube)^†^	3	16.1 (5.3)	3	21.4 (3.5)	5.3	−4.8	15.3	0.22

All values are mean (standard deviation) and expressed as percentage (%) of nominal dose (5 mg). *P < 0.05. ^†^Backcalculated data.

**Figure 2:**
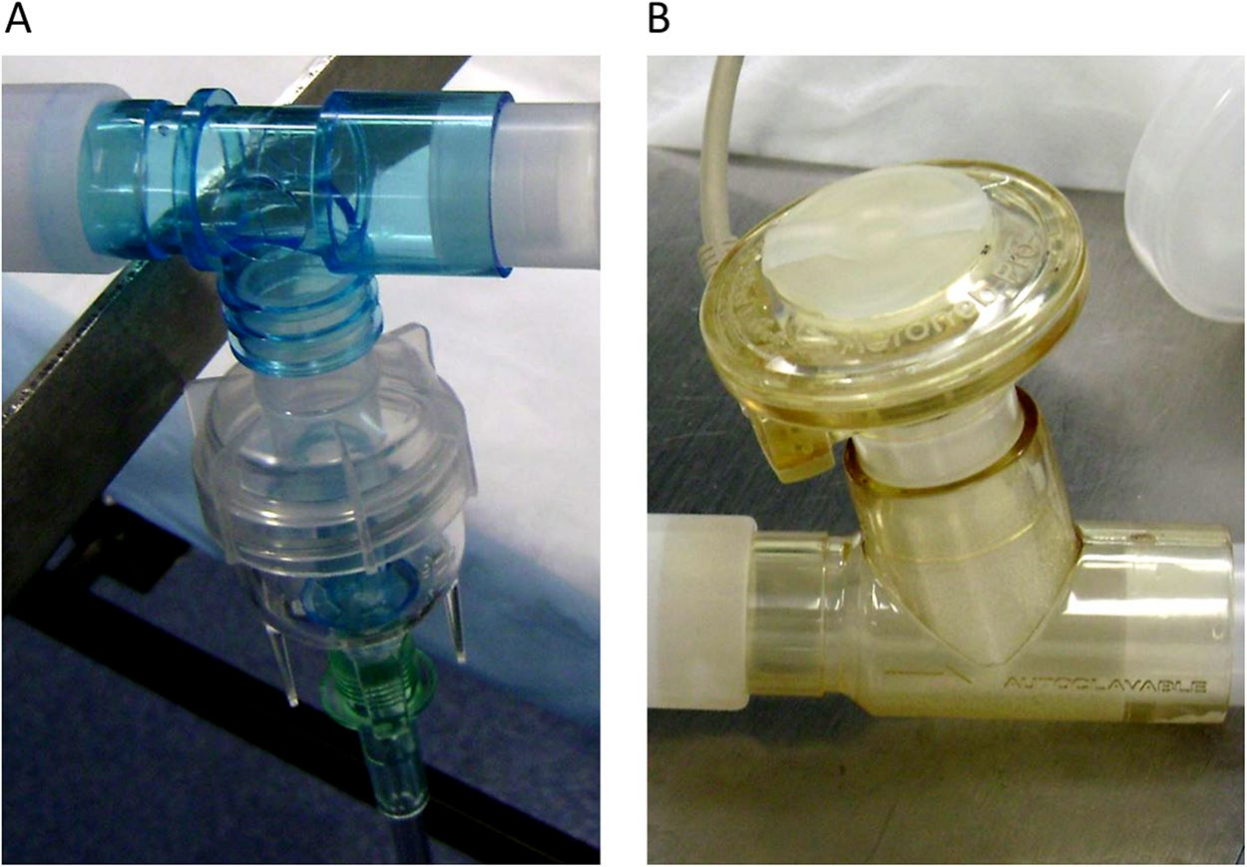
Jet nebulizer (Airlife Misty Max 10 Nebulizer; Salter Labs, Arvin, CA, USA). (**A**) Mesh nebulizer (Aeroneb Professional Nebulizer System; Aerogen Ltd., Galway, Ireland) (**B**).

*Setting 1* (Fig. 2A) was used to determine medication leaving the nebulizer (emitted dose; filter A). *Setting 2* (Fig. 2B) was used to determine medication inhaled into the patient’s lungs (inhaled dose; filter B) and medication wasted during the expiratory phase (exhaled dose; filter C).

*In Setting 2*, the mean percentage of the inhaled dose (filter B) was almost threefold higher with mesh (46.4%) compared to jet (16.6%) nebulization (mean difference, 29.8%; 95% CI, 14.1 to 45.5; *P* = 0.006). Accordingly, a lower exhaled dose (filter C) was observed with mesh (7.4%) compared to jet (34.1%) nebulization (mean difference, 26.7%; 95% CI, 19.0 to 34.4; *P* < 0.001). For both types of nebulizers, distribution of the residual dose and wasted dose within the nebulizer cup and T-piece, respectively, were similar to those observed in experiments from *Setting 1* (*P* > 0.05). The mean total dose recovered was 78.6% and 75.1% with jet and mesh nebulization, respectively (mean difference, 3.5%; 95% CI, −9.4 to 16.4; *P* = 0.49). Consequently, mean backcalculated unrecovered dose in the Y-connector and endotracheal tube was estimated as 16.1% and 21.4% with jet and mesh nebulization, respectively (mean difference, 5.3%; 95% CI, −4.8 to 15.3 *P* = 0.22).

## Discussion

This is the first study reporting on both milrinone early systemic exposure and inhaled dose after nebulization in cardiac surgical patients. In agreement with their respective *in vitro* inhaled dose, *in vivo* early plasma concentrations were almost threefold higher with mesh compared to jet nebulization.

The notion of “inhaled aerosol” was introduced for the first time in 1991 by Smaldone^24^, who defined the inhaled dose as the mass of medication ultimately inhaled into the patient’s airways. Since then, quantification of the inhaled dose in typical clinical settings and conditions has become a popular method for evaluating the performance of devices in pulmonary drug delivery^19,25^. Inhaled milrinone is still investigational, as it is not yet labeled for the use under discussion.

One of the major benefits of using nebulizers to deliver drugs to the lungs lies in their unique ability to transform drug solutions into fine, breathable droplets. However, like any pulmonary drug delivery system, their performance varies considerably depending on intrinsic factors such as brand and design, additionally to extrinsic factors such as fill volume, flow rate and position in the setting^19,23,26–28^. Furthermore, factors determining drug delivery to the lungs in presence of mechanical ventilation differ significantly from those in spontaneous breathing^23,28^. Therefore, the choice of inhalation device and appropriate administration techniques according to the type of setting will highly influence the pharmacokinetics of inhaled milrinone (e.g., inhaled dose, input rate, systemic levels, etc.), and eventually drug response, in cardiac patients. *In vitro* studies were therefore necessary for quantitative assessment of milrinone inhaled dose and proper interpretation of future pharmacokinetic data.

Our *in vitro* experiments (*Setting 1*) showed that both jet and mesh nebulizers generated similar emitted doses (Table 4). However, the residual dose in the nebulizer cup was significantly lower with the mesh nebulizer and consistent throughout experiments. Indeed, high stability and minimal residual volume are two well-known properties of mesh nebulizers^21,23,28^. It is worth mentioning that, although distributed differently within the nebulizer two components (cup and T-piece), approximately 30% of milrinone nominal dose remained trapped within the device in both settings and for both types of nebulizers. This also suggests that the presence of filters placed at different positions in the ventilator circuit did not influence devices performance. In addition, results from *Setting 2* showed that milrinone inhaled and exhaled doses together accounted for 50% of nominal dose, for both types of nebulizers. Accordingly, it was estimated that the remaining wasted dose in the ventilator circuit, including the Y-connector and endotracheal tube, would be approximately 20% of the nominal dose.

Evaluation of efficiency in drug delivery yielded an *in vitro* inhaled dose corresponding to 16.6% of milrinone nominal dose with jet nebulization and 46.4% (almost threefold higher) with mesh nebulization. Other studies testing the same brands of nebulizers have reported similar findings^21,27,28^. In mechanically ventilated patients, 2–3 fold greater inhaled drug was also observed with the same model of mesh nebulizer compared to another model of jet nebulizer placed in the same setting position^21,22,29^. From our *in vitro* experiments, the poor efficiency in delivering a higher inhaled dose with the jet nebulizer may be partially explained by a proportionally higher exhaled dose compared to the mesh nebulizer.

Since inhalation is being considered as an alternative therapeutic route of administration for milrinone in cardiac surgical patients, it was important to determine the early systemic exposure after nebulization in order to verify whether these plasma concentrations were susceptible to exceed the therapeutic range (100–300 ng·ml^−1^)^5,30,31^ and cause systemic hypotension^5–7^. In agreement with their respective inhaled dose determined during *in vitro* experiments, milrinone early systemic exposure was influenced by the type of nebulizer, which resulted in plasma concentrations 2–3 fold greater with mesh compared to jet nebulization. After administration of a 5 mg (50–80 µg·kg^−1^) dose of milrinone by inhalation using either type of nebulizer, plasma levels observed in our patients were below 100 ng·ml^−1^, and well below those reported after a 50 μg·kg^−1^ intravenous bolus dose of milrinone (over 600 ng·ml^−1^)^32^. No systemic hypotension was reported in our patients. Significant reduction in blood pressure or mAP following the administration of milrinone has been described when using intravenous milrinone. This is most likely related to the systemic vasodilatory effect of the drug and the elevated peak plasma concentrations (over 200 ng·ml^−1^)^32,33^. However, when milrinone is administered by inhalation, plasma levels are lower and no systemic hypotension is reported (as demonstrated in this study)^34–36^. In a recent multicenter randomized controlled trial, we did not observe any difference in terms of arterial pressure change in the group receiving inhaled milrinone vs. placebo^17^. Furthermore, different hemodynamic effects suggesting selective pulmonary vasodilation and increased atrial contraction were observed^17^.

The use of a relative hemodynamic parameter to evaluate the severity of PH such as the mAP/mPAP ratio (normal 4 to 1) has been shown to be much more predictive of outcome in various types of cardiac surgical procedures^37–41^. Induction of anesthesia reduces all pressures including mAP and mPAP, but the mAP/mPAP ratio remains unchanged^37^. As right ventricular dysfunction develops with PH, the absolute pulmonary artery pressures will tend to normalize but the ratio will remain abnormal. In our study, although there was a significant difference in baseline values for mAP between groups (82.6 mmHg vs. 67.4 mmHg for jet and mesh, respectively), this was corrected by using the mAP/mPAP ratio, which baseline values were not significantly different between groups before drug administration (3.0 vs. 2.6 for jet and mesh, respectively). This ratio is used as a pharmacodynamic measurement of the effect of inhaled pulmonary vasodilators^17^. For instance, if a vasodilator is active and selective to the pulmonary circulation, mAP will remain unchanged but mPAP will decrease and the mAP/mPAP ratio will increase. On the other hand, if a non-selective vasodilator is administered, both mAP and mPAP will decrease but the mAP/mPAP ratio will remain unchanged. In this study, the administration of milrinone significantly reduced mPAP by 26.2% (−7.1 mmHg) and increased the mAP/mPAP ratio by 32.1% (0.8) after mesh nebulization compared to baseline values. These hemodynamic improvements were not statistically significant after jet nebulization. Therefore, the systemic blood levels generated by inhaled milrinone, although not associated with systemic hypotension, may reflect local pulmonary levels that are effective for reducing the severity of PH as its site of action is located within the pulmonary vascular smooth muscle cells. In other words, our results suggest that it is unlikely that either of the two types of nebulizers would generate sufficiently high plasma levels to induce systemic hypotension in cardiac surgical patients, but mesh nebulization may provide better efficiency in delivering milrinone to the lungs and potentially improve the efficacy of PH treatment.

Because delivering drug to the lungs is a challenging process depending on numerous clinical and device-related factors^19,23,26–28^, *in vitro* testing can provide the end user with significant information and guidance on appropriate administration techniques for optimal devices performance. In order to acquire useful insight into the pulmonary deposition pattern of inhaled milrinone, our team studied the *in vitro* particle size distribution using both jet and mesh nebulization^42^. Gavra *et al*. found more milrinone particles in the lower airways (mean aerodynamic diameter from 1.4 to 5.4 μm) with mesh nebulization, while greater milrinone particles were collected from the higher airways (mean aerodynamic diameter over 14.1 μm) with jet nebulization. However, milrinone optimal site of deposition in the lungs after inhalation remains unclear. In that study, the inhaled dose was reported as 30% and 60% with jet and mesh nebulization, respectively. Differences in their animal experimental setting and breathing patterns could explain the higher inhaled doses observed compared to those obtained from our *in vitro* experiments (17% and 46% for jet and mesh, respectively): breathing simulation with single input/output port, constant negative pressure, higher respiratory rate, lower minute volume and lower temperatures (7 °C).

### The present study has important limitations

The main one, which constitutes the purpose of additional studies, consists of the absence of a full characterization of milrinone pharmacokinetics and *in vivo* inhaled dose. The duration of nebulization was very variable and not precisely documented *in vivo*, which explains why exact timing of blood sampling after inhalation was approximate and may not always represent milrinone maximum concentration. Importantly, the main purpose of this study was to provide preliminary data for a full-scale study on the concentration-effect relationship of inhaled milrinone in cardiac surgical patients.

In conclusion, adequate administration of inhaled drugs relies on careful evaluation of aerosol devices, delivery systems and dosing. Low plasma concentrations of milrinone are observed in cardiac surgical patients following both jet and mesh nebulization. However, mesh nebulization provides better efficiency in delivering aerosolized milrinone to mechanically ventilated patients, resulting in almost threefold increased inhaled dose and systemic exposure compared to conventional jet nebulization. The vibrating mesh nebulizer is more suitable for clinical setting; it offers continuous aerosolized drug therapy, does not require a secondary airflow with additional oxygen which could alter the severity of PH, is reusable, compact and portable compared to the simple jet nebulizer. These data suggest that inhalation represents a promising alternative route of administration for milrinone in cardiac surgery in order to avoid systemic hypotension, and warrants further investigations in a larger scale study.

## Materials and Methods

### Study design and patients

Following permission from Health Canada (non-objection letter, ref. 108851; November 2, 2006), the study was approved by the Montreal Heart Institute Research Ethics Committee (ref. ICM 06-888; December 6, 2006). The present study is an exploratory substudy (pilot study) of the main research protocol later amended (ref. ICM 06-888; August 5, 2008) and registered at ClinicalTrials.gov (NCT01725776). Written informed consent was obtained from a convenience sample of 12 patients diagnosed with preoperative PH and scheduled for elective cardiac surgery using CPB. Preoperative PH was defined as either systolic pulmonary artery pressure (sPAP) > 35 mmHg or mPAP > 25 mmHg prior to surgery^43^. Patients with preoperative hemodynamic instability, defined as acute requirement for vasoactive or mechanical support prior to surgery, were excluded. All methods were performed in accordance with the relevant guidelines and regulations.

### Surgical protocol

Patients were premedicated with lorazepam (1–2 mg orally) one hour before surgery, morphine (0.1 mg·kg^−1^ intramuscularly) before entering the operating room and midazolam (0.01–0.05 mg·kg^−1^ intravenously) at the discretion of the anesthesiologist. In addition to routine monitors^44^, a five-lead electrocardiogram, radial and femoral arterial catheters, central venous pressure catheter, and a fast-response thermodilution pulmonary artery catheter were placed before induction of anesthesia. Anesthesia was induced with sufentanil (1 μg·kg^−1^ intravenously) and midazolam (0.04 mg·kg^−1^), and muscle relaxation was achieved with pancuronium (0.1 mg·kg^−1^ intravenously). After tracheal intubation, anesthesia was maintained with sufentanil (1 μg·kg^−1^·h^−1^) and midazolam (0.04 mg·kg^−1^·h^−1^). Hemodynamic monitoring post-induction of anesthesia included mAP, mPAP and the mAP/mPAP ratio before starting inhalation (baseline; 0 min) and after the start of inhalation (at 15, 20, 25, 30, 35, 40, 45 min). Cardiopulmonary bypass was instituted using arterial cannulation of the distal ascending aorta and venous (two-stage or bicaval) cannulation of the right atrium. Blood to crystalloid (4:1) cardioplegia was administered intermittently during CPB with induction and maintenance temperatures ranging from 15 to 29 °C. The patient’s systemic temperature was allowed to drift to 34 °C for coronary artery bypass procedures, and to 32–34 °C for valve and more complex procedures. Weaning from CPB commenced after rewarming to a systemic temperature > 36 °C.

### Drug administration

The day before surgery, eligible patients were randomized to receive milrinone by inhalation using either a jet (Airlife Misty Max 10 Nebulizer; Salter Labs, Arvin, CA, USA) or a mesh nebulizer (Aeroneb Professional Nebulizer System; Aerogen Ltd., Galway, Ireland). Randomization was achieved according to a list of random numbers that were assigned to the study devices by a research coordinator who conducted patient recruitment and allocation sequence. The investigators had no access to the randomization list until after data analysis. After induction of anesthesia and baseline transesophageal echocardiography examination, 5 mg (50–80 µg·kg^−1^) of milrinone (Milrinone Lactate 1 mg·ml^−1^; Pharmaceutical Partners of Canada Inc., Richmond Hill, ON, CAN) was administered by inhalation before initiation of CPB. Jet nebulization was achieved using a secondary source of airflow (8 L·min^−1^) from wall oxygen, while mesh nebulization was performed using a battery-powered controller. The assigned nebulizer was connected into the inspiratory limb of the ventilator circuit, immediately before the Y-connector and the endotracheal tube. Milrinone nominal dose, defined as the total drug dose placed in the nebulizer cup, was nebulized until aerosol production was deemed complete (typically 15–20 minutes) after gentle tapping and visualization of the device (for both types of nebulizers). Concomitant medications were administered according to local standards of care, with the exception of milrinone.

### *In vivo* early systemic exposure

Blood samples were drawn to study milrinone early systemic exposure. Arterial blood samples (5 ml) were collected in lithium heparin tubes (Vacuette^®^ heparin tubes; Grenier Bio-One, Kremsmünster, Austria) before starting inhalation (baseline; 0 min) and after the start of inhalation (at 15, 20, 25, 30 min). Samples were kept on ice for a short period of time before centrifugation (3500 rpm; 15 min; 4 °C), and plasma was immediately flash-frozen on dry ice for storage at −80 °C until analysis. Milrinone plasma concentrations were determined by high-performance liquid chromatography using ultraviolet detection (HPLC-UV), as previously described^35^. The lower limit of quantification (LLOQ) was 1.25 ng·ml^−1^ with mean intra-assay (n = 6) and inter-assay (n = 5) precisions of < 8%, expressed as coefficients of variation (CV%).

### *In vitro* inhaled dose

This part of the study was designed to closely replicate the typical clinical setting and conditions in the operating room. On three different days and for each type of nebulizer, experiments that mimic *in vivo* administration of inhaled milrinone to cardiac surgical patients were carried out to determine devices performance in terms of emitted dose and inhaled dose. The experimental setup (breathing system) consisted of a disposable adult breathing circuit connected to an anesthesia workstation (7000 Anesthesia Ventilator Multi-Voltage Electronic; Ohmeda, Madison, WI, USA) with an expandable balloon (3.0 L Rusch^®^ Breathing Bag; Teleflex, Morrisville, NC, USA) placed at the patient interface (i.e., directly connected to the distal end of the endotracheal tube). Representative breathing patterns for our patient population were used including tidal volume (500 ml), respiratory rate (12 breaths per minute), minute volume (6 L per minute), and inspiratory:expiratory ratio (1:2)^45,46^.

**Figure 3:**
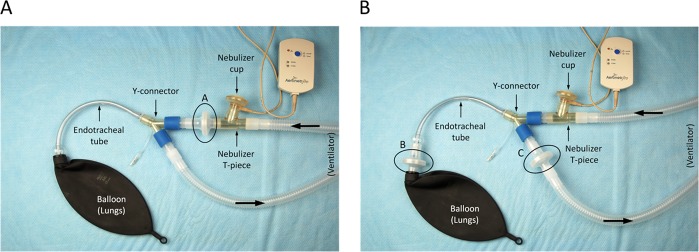
*In vitro* settings for estimation of milrinone dose recovery (mesh nebulizer displayed). *Setting 1* (**A**) was used to determine the emitted dose (filter **A**). *Setting 2* (**B**) was used to determine the inhaled dose (filter **B**) and exhaled dose (filter **C**). The residual dose (nebulizer cup) and wasted dose (nebulizer T-piece) were also measured.

Each nebulizer was composed of a cup (reservoir) and a T-piece. The jet nebulizer was designed with the T-piece positioned on top of the nebulizer cup (Fig. 2A) and *vice versa* for the mesh nebulizer (Fig. 2B). Low-resistance collecting filters (Vital Signs Inc., Totowa, NJ, USA) were used to collect aerosolized milrinone over the duration of nebulization (Fig. 3). For each type of nebulizer, two series of experiments were carried out using the same setup, but having collecting filters placed at different positions within the breathing circuit (Fig. 3A,B). Milrinone administration was as earlier described for patients.

*Setting 1* (Fig. 3A) was used to determine the emitted dose, defined as the mass of medication leaving the nebulizer. For this experiment, filter A (emitted dose) was placed immediately at the outflow of the nebulizer T-piece. *Setting 2* (Fig. 3B) was used to determine the inhaled dose and exhaled dose, defined as the mass of medication inhaled into the patient’s lungs and the mass of medication wasted during the expiratory phase, respectively. For this experiment, filter B (inhaled dose) was placed at the distal end of the endotracheal tube immediately before the expandable balloon (3.0 L breathing bag), and filter C (exhaled dose) was placed at the expiratory limb of the ventilator circuit immediately after the Y-connector.

At the end of nebulization, the doses remaining within the nebulizer cup (residual dose) and the nebulizer T-piece (wasted dose) were determined. Laboratory film (Parafilm^®^; Bermis Company, Inc., Neenah, WI, USA) was used to seal the T-piece and each of the nebulizer components (cup and T-piece) was rinsed with sterilized water (2 ml) before collection in a test tube for analysis. Milrinone content was eluted from filters after immersion in 250 ml buffer solution (NaH_2_PO_4_ 50 mM, pH 3) and 10 min ultrasound sonication (Ultrasonic Cleaner FS 28 H; Fisher Scientific Co., Hampton, NH, USA). Preliminary experiments had confirmed quantitative recovery of milrinone from filters (99.1%, n = 4). Samples were stored at −20 °C until analysis. Milrinone concentrations were determined by HPLC-UV using a simplified version of the assay used for plasma (see above). Six concentrations of milrinone prepared in buffer solution (20 down to 0.01 µg·ml^−1^) were used to establish calibration curves (r^2^ = 0.9967, n = 6). Each sample was injected twice in the HPLC with the mean value used for data analysis.

### Statistical analysis

For the *in vivo* experiments, patient characteristics and milrinone concentrations at each time point were expressed as mean (standard deviation). Comparisons between groups (jet vs. mesh) were performed using unpaired Student t-test for continuous variables and Chi-square test for categorical variables. Comparisons within group (post-inhalation vs. baseline) were performed using paired Student t-test.

For the *in vitro* experiments, mean individual dose recovered was determined and expressed as a percentage of the nominal dose (5 mg). For each replicate, the total dose recovered was obtained by summing individual recoveries. In *Setting 2* only, unrecovered doses in the Y-connector and the endotracheal tube could not be directly determined due to the difficulty of disconnecting these components without significant spillage. Instead, the unrecovered dose from those two components was backcalculated by subtracting individual recoveries determined for the other components from the total dose recovered determined in *Setting 1*.

Statistical analysis was carried out with SigmaPlot^®^ version 11.0 (Systat Software, Inc., San Jose, CA, USA). A *P* < 0.05 was considered statistically significant.

## Data Availability

All data generated or analyzed during this study are available from the Correspondings Authors.
